# ***Candida albicans*** **is a context-dependent risk factor for malignant transformation of oral precancer lesions: a prospective cohort study of 734 Taiwanese patients**

**DOI:** 10.1080/20002297.2025.2598743

**Published:** 2025-12-17

**Authors:** Shih Sheng Jiang, Chung-Hsing Chen, Fang-Yu Tsai, Yi-Ping Hsieh, Tsung-Te Chung, Jang-Jaer Lee, Mu-Kuan Chen, Yen-Tze Liu, Shun-Fa Yang, Chun-Yi Chuang, Wen-Lun Wang, Chih-Chun Wang, Tze-Ta Huang, I-Chi Chen, Pei-Hua Wu, Yi-Chieh Chen, Ya-Wen Chen, Shine-Gwo Shiah, Li-Hsin Chien, I-Shou Chang, Ching-Yu Yen, Ko-Jiunn Liu

**Affiliations:** a National Institute of Cancer Research, National Health Research Institutes, Miaoli, Taiwan; b Institute of Basic Medical Sciences, College of Medicine, National Cheng Kung University, Tainan, Taiwan; c Department of Otolaryngology, Show Chwan Memorial Hospital, Changhua, Taiwan; d Department of Dentistry, National Taiwan University Hospital, Taipei, Taiwan; e Department of Dentistry, National Taiwan University College of Medicine, Taipei, Taiwan; f Department of Otorhinolaryngology-Head and Neck Surgery, Changhua Christian Hospital, Changhua, Taiwan; g Graduate Institute of Clinical Medicine, College of Medicine, National Chung Hsing University, Taichung, Taiwan; h Department of Post-Baccalaureate Medicine, College of Medicine, National Chung Hsing University, Taichung, Taiwan; i Oral Cancer Research Center, Changhua Christian Hospital, Changhua, Taiwan; j Institute of Medicine, Chung Shan Medical University, Taichung, Taiwan; k Department of Medical Research, Chung Shan Medical University Hospital, Taichung, Taiwan; l Department of Otolaryngology, Chung Shan Medical University Hospital, Taichung, Taiwan; m School of Medicine, Chung Shan Medical University, Taichung, Taiwan; n Department of Internal Medicine, Division of Gastroenterology and Hepatology, E-Da Hospital/I-Shou University, Kaohsiung, Taiwan; o School of Medicine, College of Medicine, I-Shou University, Kaohsiung, Taiwan; p College of Medicine, I-Shou University, Kaohsiung, Taiwan; q Department of Otolaryngology, E-Da Hospital, Kaohsiung, Taiwan; r School of Dentistry, and Institute of Oral Medicine, National Cheng Kung University, Tainan, Taiwan; s Department of Stomatology, National Cheng Kung University Hospital, Tainan, Taiwan; t Department of Applied Mathematics, National Dong Hwa University, Hualien, Taiwan; u School of Dentistry, Taipei Medical University, Taipei, Taiwan; v Department of Dentistry, Chi Mei Medical Center, Tainan, Taiwan

**Keywords:** Oral potentially malignant disorders, oral squamous cell carcinoma, *Candida albicans*, malignant transformation, oral microbiota

## Abstract

**Background:**

*Candida albicans* has been implicated in oral carcinogenesis, but its role in the progression of oral potentially malignant disorders (OPMDs) remains unclear. We investigated whether high *Candida* burden in OPMD lesions predicts malignant transformation (MT) and whether this association varied by OPMD subtype.

**Patients and methods:**

In a multicenter prospective cohort study across seven hospitals in Taiwan, 734 OPMD patients were followed for a mean of 2.4 years. Oral lesion swabs were cultured on chromogenic agar to quantify *Candida albicans* level. Cox models were used to estimate hazard ratios (HRs) for MT to oral cancer.

**Results:**

MT occurred in 6.8% of patients. High *Candida* burden was independently associated with increased MT risk (aHR = 2.84; 95% CI: 1.40–5.75). Patients with oral submucous fibrosis (OSF) or verrucous hyperplasia (VH) also had elevated risk (aHR = 4.99; 95% CI: 1.54–10.38). Interaction analysis revealed strong individual risks for high *Candida* burden (aHR = 13.83) and OSF/VH (aHR = 13.67), with an attenuating interaction term (aHR = 0.11), yielding a substantial combined risk (HR ≈ 20.8). Stratified analysis showed the strongest effect in leukoplakia (HR = 12.19).

**Conclusions:**

High *Candida albicans* burden is a significant, subtype-dependent risk factor for malignant progression in OPMDs. These findings underline the role of fungal–host interactions in oral carcinogenesis and support the integration of fungal profiling into routine surveillance of OPMDs.

## KEY MESSAGES

High oral *Candida albicans* burden is an independent risk factor for malignant transformation among patients with oral potentially malignant disorders.

The prognostic impact of *Candida albicans* is context-dependent, with the strongest effect observed in lower-risk epithelial lesions such as leukoplakia.

Quantifying fungal burden in the oral cavity may improve personalized surveillance strategies for early detection and prevention of oral cancer.

## GRAPHICAL ABSTRACTS

**Figure:**
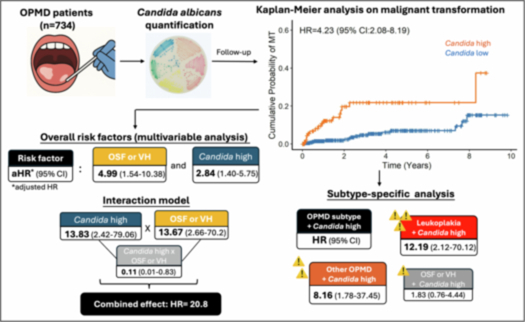

## Introduction

Oral cancer represents a significant global health challenge, with oral squamous cell carcinoma (OSCC) constituting the majority of cases [1]. In 2020, the Global Cancer Observatory reported over 389,000 new cases and 188,000 associated deaths worldwide, predominantly in Asia [1,2]. OSCC is a male-predominant malignancy, with a male-to-female incidence ratios ranging from 2.2-2.8:1 across regions of varying socioeconomic development [2]. In Taiwan, OSCC ranks as the fourth most common cancer among males [3,4]. Prominent risk factors for OSCC include alcohol consumption, betel quid chewing, and cigarette smoking, which are implicated in the multifactorial aetiology of the disease through both genetic and epigenetic modifications [1,5–8].

Oral potentially malignant disorders (OPMD), such as leukoplakia, erythroplakia, oral submucous fibrosis (OSF), lichen planus, and verrucous hyperplasia (VH), are well-recognised precursors to OSCC [9]. Among these, OSF and VH exhibit the highest rates of malignant transformation (MT) [10–14]. Additionally, dysplasia remains the most critical histopathological predictor of progression [12,15–17]. However, transformation rates vary widely across OPMD subtypes, complicating risk stratification and early detection strategies. Notably, recent attention has turned to microbial factors, particularly *Candida albicans*, as potential contributors to this MT process, a gap that remains insufficiently explored.

While often a harmless commensal of the oral microbiota, *C. albicans* can undergo pathogenic transitions under dysbiotic or immunocompromised conditions [13,18–20]. This shift may lead to biofilm formation, mucosal invasion, and release of pro-inflammatory and carcinogenic metabolites, including nitrosamines and acetaldehyde [21–23]. These compounds can cause DNA damage and contribute to oncogenic transformation. Although prior studies have linked *C. albicans* to OSCC progression [24,25], its specific contribution to the malignant transformation of OPMDs remains unclear.

This study investigates whether a high oral burden of *Candida albicans* is associated with increased risk of malignant transformation in a prospective multicenter cohort of Taiwanese patients with OPMDs.

## Methods

### Study design and population

This multicenter, prospective cohort study enroled 806 patients clinically diagnosed OPMD from seven healthcare facilities across Taiwan between February 2015 and September 2023. Participating centres included five medical centres: National Taiwan University Hospital, Chung Shan Medical University Hospital, Changhua Christian Hospital, National Cheng Kung University Hospital, Chi Mei Medical Centre, along with one quasi-medical centre (E-Da Hospital) and one regional hospital (Show Chwan Memorial Hospital).

Eligible participants were aged 20 to 80 years, had no previous cancer diagnosis, and presented with one or more OPMD subtypes, including oral leukoplakia, erythroleukoplakia, erythroplakia, lichen planus, OSF, VH, or other persistent oral mucosal abnormalities. The latter was operationally defined as persistent intraoral or perioral swellings of unknown origin, non-healing ulcers or erosions lasting more than two weeks, or mucosal surface changes (e.g. discoloration, thickening) without a definitive diagnosis.

Exclusion criteria included any history of invasive cancer, known immunocompromised status (e.g. HIV infection, organ transplantation, or ongoing systemic immunosuppressive or corticosteroid therapy), or use of systemic or topical antifungal agents prior to oral swab collection. All participants were confirmed to be antifungal-naïve at baseline, which served as a key inclusion requirement.

At baseline, participants underwent a standardised oral examination performed by board-certified oral medicine specialists. Structured interviews were conducted to collect data on demographics, health behaviours (smoking, alcohol, and betel quid use), and clinical status. Oral swabs were obtained from both lesion and non-lesion sites to quantify *Candida albicans* burden (cf. Detection and Quantification of Oral *Candida albicans* Level section).

Participants were followed every six months through routine clinical surveillance to monitor for progression to OSCC, with diagnoses confirmed histologically. Follow-up continued until MT, loss to follow-up, or study end. The study closed on December 31, 2024. All participants enroled before December 31, 2023, had at least 12 months of follow-up. The cohort, referred to as TWOPMD, was designed and reported in accordance with the STROBE (STrengthening the Reporting of OBservational Studies in Epidemiology) guidelines. Of the 806 enroled individuals, 72 were excluded from the longitudinal analysis: 8 who were diagnosed with OSCC within 30 days of enrolment and 64 who were lost to follow-up before their first scheduled visit. These exclusions were applied post-enrolment but prior to outcome ascertainment to ensure that all included participants were at risk and adequately monitored. The remaining 734 individuals comprised the TWOPMD-1 sub-cohort for longitudinal follow-up.

Of the 734 participants included in the final analytic cohort, 50 developed malignant transformation (MT) during the study period. The remaining 684 participants were censored at the end of follow-up on December 31, 2024, as they remained free of MT and none were lost to follow-up. Thus, no participants were censored due to loss to follow-up, and no imputation for missing survival outcome data was necessary. Time-to-event was calculated from the date of enrolment to either histologically confirmed MT or the study end date, whichever came first.

### Detection and quantification of oral *Candida albicans* level

To determine oral *Candida albicans* burden, swabs were collected from all lesion sites of each patient using sterile cotton swabs, applying a single swabbing motion per lesion. Additionally, one swab from a non-lesion mucosal site was obtained for each participant, typically from a clinically healthy area distant from any lesion (e.g. the contralateral buccal mucosa). As some patients presented with multiple lesions, the number of lesion-site swabs varied. Swabs were immediately placed on ChromID™ Candida Agar (bioMérieux, France) and stored at 4°C. After 48-hour incubation at 33–37°C, *Candida albicans* colonies were identified by characteristic blue pigmentation. Colony-forming units (CFU) were counted from high-resolution images, with two independent reviewers confirming results. The highest CFU count across all sampled sites (including lesion and non-lesion sites) was used as the individual’s representative fungal burden, because in many cases, *Candida* growth at the non-lesion site was often comparable to or occasionally even higher than that observed at lesion sites (Supplementary Table S1). Confirmation of *C. albicans* presence was validated using an RT-PCR method, as detailed in the Supplementary Methods, adapted from the protocol described by Guiver et al. [26].

### Definition of study variables

Sociodemographic and behavioural data were obtained via structured, face-to-face interviews. Variables were defined as follows. Alcohol consumption and betel quid chewing were categorised into never, occasional/former, and current, while tobacco smoking into never, ever, and current. An ‘ever’ smoker is one who smoked more than 100 cigarettes during their lifetime. Multifocal lesion was defined as multiple lesions at distinct oral sites, while a unifocal lesion means a lesion at single site. Multiform lesion was defined as more than one OPMD subtype present, while a patient presenting only one subtype was termed uniform. The fibrosis severity was defined based on test to measure the length of the mouth opening, categorised as follows: none (>35mm), mild (26-35mm), moderate (15-25mm), and severe (<15mm). The epithelial dysplasia was determined based on the histopathological images from the major lesion and categorised as no dysplasia, dysplasia present, or unknown due to no biopsy performed. The levels of *Candida albicans* colonisation were dichotomised as low (0-50 CFU) or high (>51 CFU).

### Statistical analysis

Time-to-event outcomes were analysed using Cox proportional hazards models. The proportional hazards assumptions were assessed using Schoenfeld residuals and found to be satisfied. *Candida* burden data were missing for approximately 25% of eligible participants. Rather than excluding these individuals from the primary analysis, we retained them as a separate ‘unknown’ category to preserve sample size and allow comparative analysis. Given indications of non-random missingness (data not shown), we did not perform data imputation, as it could introduce bias under likely missing-not-at-random (MNAR) conditions. Multivariable model predictors were selected using the Akaike Information Criterion (AIC) [27]. To evaluate potential multicollinearity, Variance Inflation Factor (VIF) [28,29] was calculated. To explore the associations between *Candida albicans* burden and lesion subtypes, we conducted two logistic regression analyses: one with *Candida* level as the outcome and lesion subtype as the predictor, and another reversing this structure. All analyses were conducted using R software version 4.3.1. The packages used include ‘*survival’* for Cox proportional hazards modelling, ‘*MASS’* for stepwise variable selection using AIC, and ‘*car’* for collinearity diagnostics (Variance Inflation Factor).

## Results

### Demographics, clinical characteristics, and *Candida albicans* levels in TWOPMD-1 cohort

Of the 806 participants prospectively enroled in the TWOPMD cohort, 72 were excluded dude to a diagnosis of OSCC within 30 days of enrolment (*n* = 8) or lost to follow-up prior to the first scheduled visit (*n* = 64), leaving 734 eligible for longitudinal analysis (TWOPMD-1 sub-cohort). We compared baseline characteristics between included (*n* = 734) and excluded (*n* = 72) patients (Supplementary Table S2). The groups were broadly similar in terms of age, sex, education level, lifestyle behaviours (alcohol consumption, betel quid chewing, and cigarette smoking), oral *Candida albicans* levels, and lesion diversity. These similarities suggest that missingness was likely random and unlikely to introduce meaningful selection bias.

Table 1 summarises demographic, lifestyle, and clinical variables. The cohort was predominantly male (89.8%), with a mean age of 52.3 years (SD: 11.5). Educational attainment varied: 40.8% had primary school education, 16.9% completed high school, and 42.3% had a college-level education. A majority of participants reported histories of alcohol consumption (82.3%), betel quid chewing (78.4%), and cigarette smoking (85.6%).

**Table 1.** Demographic, lifestyle, and clinical characteristics of the TWOPMD-1 cohort.

Variable	No MT (%)	MT to OSCC (%)	*p-*value*	Total (%)
Number of patients				
	684 (93.2)	50 (6.8)		734
Age (year)			**0.031** ^#^	
Mean (SD)	52.1 (11.6)	55.1 (9.0)		52.3 (11.5)
Follow-up (day)			**0.080** ^#^	
Mean (SD)	899.8 (961.1)	702.1 (741.3)		886.4 (948.6)
Gender			0.816	
Male	610 (93.1)	45 (6.9)		655 (89.8)
Female	70 (94.6)	4 (5.4)		74 (10.2)
Education			0.05	
Primary school	269 (90.6)	28 (9.4)		297 (40.8)
High school	118 (95.9)	5 (4.1)		123 (16.9)
College	292 (94.8)	16 (5.2)		308 (42.3)
Alcohol drinking			0.849	
Never	119 (92.2)	10 (7.8)		129 (17.7)
Occasional + Former	285 (93.8)	19 (6.2)		304 (41.8)
Current	275 (93.2)	20 (6.8)		295 (40.5)
Betel quid chewing			0.256	
Never	151 (96.2)	6 (3.8)		157 (21.6)
Occasional + Former	352 (92.4)	29 (7.6)		381 (52.4)
Ever	175 (92.6)	14 (7.4)		189 (26.0)
Cigarette smoking			0.873	
Never	97 (92.4)	8 (7.6)		105 (14.4)
Ever	127 (94.1)	8 (5.9)		135 (18.6)
Current	454 (93.2)	33 (6.8)		487 (67.0)
Lesion focality			**0.006**	
Unifocal	278 (96.5)	10 (3.5)		288 (39.2)
Multifocal	406 (91)	40 (9)		446 (60.8)
Lesion diversity			**0.001**	
Uniform	458 (95.6)	21 (4.4)		479 (65.3)
Multiform	225 (88.6)	29 (11.4)		254 (34.7)
Fibrosis (mouth opening)			**0.042**	
No	481 (94.9)	26 (5.1)		507 (69.2)
Mild	113 (91.1)	11 (8.9)		124 (16.9)
Moderate	31 (83.8)	6 (16.2)		37 (5.0)
Severe	10 (90.9)	1 (9.1)		11 (1.5)
Unknown	48 (88.9)	6 (11.1)		54 (7.4)
Dysplasia			**0.001**	
No	124 (97.6)	3 (2.4)		127 (17.4)
Yes	404 (90.4)	43 (9.6)		447 (61.2)
No biopsy	152 (97.4)	4 (2.6)		156 (21.4)
Oral *Candida* level			**2.5×10^−4^**	
Low (0-50 CFU)	416 (96.1)	17 (3.9)		433 (59.0)
High (>50 CFU)	98 (86)	16 (14)		114 (15.5)
(Unknown)	170 (90.9)	17 (9.1)		187 (25.5)
Major lesion subtype			**3.5×10^−5^**	
Only leukoplakia**^§^**	333 (96.8)	11 (3.2)		344 (46.9)
OSF/VH	195 (87.1)	29 (12.9)		224 (30.5)
Others**^¶^**	156 (94)	10 (6)		166 (22.6)
Treatment modality			0.112	
No medication/CO_2_ laser	157 (96.3)	6 (3.7)		163 (22.4)
Biopsy/reconstruction	521 (92.4)	43 (7.6)		564 (77.6)

^*^p-values are from Chi-square test unless otherwise specified; *p* < 0.05 are in bold.

^#^ *p*-values from *t*-tests.

^§^ Patients with leukoplakia (uniform) only.

^¶^ Patients not in the category of ‘only leukoplakia’ or ‘OSF/VH’.

Clinically, 60.8% of patients presented with multifocal lesions, and 34.7% had multiform lesions. The most common OPMD subtypes were leukoplakia (46.9%), while 30.5% of patients had OSF or VH, both considered high-risk subtypes. Dysplasia was present in 61.2% of patients, absent in 17.4%, and unassessed in 21.4% due to unavailable biopsy samples.

Quantification of *Candida* colonisation among the cohort revealed that 433 patients (59.0%) had low colonisation, 114 (15.5%) had high colonisation, and 187 (25.5%) had unknown status.

Over a mean follow-up of 2.4 (SD, 2.6) years, 50 patients (6.8%) developed OSCC. The occurrence of MT to OSCC varied across clinical and lifestyle factors. OSCC occurred in 9% of patients with multifocal lesions compared to 3.5% with unifocal lesions, and in 11.4% of those with multiform lesions *versus* 4.4% with uniform lesions. Similarly, dysplastic lesions were more likely to progress (9.6%) than non-dysplastic ones (2.4%). Regarding OPMD subtype, the highest OSCC risk was observed in patients with high-risk subtypes (OSF/VH), at 12.9%, *versus* 3.2% in those with leukoplakia alone. In terms of *Candida* burden, 14.0% of participants with high oral *Candida albicans* levels developed OSCC, versus 3.9% of those with lower levels. These unadjusted comparisons were used to identify candidate predictors and are followed by multivariable Cox proportional hazards models to evaluate independent associations with time to OSCC, adjusting for potential confounders.

### Oral *Candida albicans* load associates with MT in patients with OPMD

Cox proportional hazards models were used to evaluate associations between clinical, microbial, and lifestyle factors and the risk of MT in the TWOPMD-1 cohort. As shown in the left panel of Table 2, univariate Cox regression revealed that high oral *Candida* burden was strongly associated with progression to OSCC. Patients with high fungal load (>50 CFU) were nearly five times more likely to undergo MT compared to those with low-level *Candida* colonisation (crude HR = 4.10; 95% CI, 2.07–8.13; *p* < 0.001). Lesion characteristics were also showed strong associations with MT risk. Multiform lesions (crude HR = 2.30; 95% CI: 1.31–4.03; *p* = 0.004) and multifocal lesions (crude HR = 2.04; 95% CI: 1.02–4.08; *p* = 0.045) both conferred higher risk. Similarly, moderate-to-severe fibrosis (crude HR = 2.09; 95% CI: 1.20–3.64; *p* = 0.01) and the presence of dysplasia (crude HR = 3.59; 95% CI: 1.11–11.59; *p* = 0.032) were significant predictors. Among lesion subtypes, patients diagnosed with OSF or VH had a fourfold increased risk of MT compared with those with leukoplakia alone (crude HR3.73; 95% CI: 1.86–7.46; *p* < 0.001). Of the lifestyle factors assessed, only betel quid chewing showed a suggestive association (crude HR = 2.28; 95% CI: 0.88–5.93; *p* = 0.091); neither alcohol consumption nor cigarette smoking were significantly associated with MT.

**Table 2.** Cox regression analysis of factors associated with MT in OPMD.

Model	Univariate		Multivariable^#^	
Variable	HR (95% CI)	*p*-value	HR (95% CI)	*p*-value
Age (year)				
	1.02 (0.99-1.05)	0.114		
Education				
Primary school	Reference			
High school	0.57 (0.31-1.05)	0.071		
College	0.47 (0.18-1.22)	0.12		
Alcohol drinking				
Never	Reference			
Occasional/Former	0.82 (0.38-1.77)	0.615		
Current	0.91 (0.42-1.94)	0.803		
Betel quid chewing				
Never	Reference			
Occasional/Former	1.85 (0.77-4.45)	0.171		
Current	2.28 (0.88-5.93)	0.091		
Cigarette smoking				
Never	Reference			
Ever	0.72 (0.27-1.93)	0.52		
Current	0.83 (0.38-1.8)	0.64		
Lesion focality				
Unifocal	Reference		Reference	
Multifocal	2.04 (1.02-4.08)	**0.045**	2.25 (1.04-4.90)	**0.045**
Lesion diversity				
Uniform	Reference		Reference	
Multiform	2.3 (1.31-4.03)	**0.004**	0.57 (0.25-1.31)	0.185
Fibrosis				
No	Reference			
Yes	2.09 (1.2-3.64)	**0.01**		
Dysplasia				
No	Reference		Reference	
Yes	3.59 (1.11-11.59)	**0.032**	2.87 (0.86-9.60)	0.087
No biopsy	0.76 (0.17-3.38)	0.713	0.63 (0.14-2.90)	0.556
Oral *Candida* level				
Low (0-50 CFU)	Reference	**0.04**	Reference	
High (>51 CFU)	4.1 (2.07-8.13)	**5.1 × 10^−5^**	2.84 (1.40-5.75)	**0.004**
Unknown	2.53 (1.29-4.96)	**0.007**	2.69 (1.37-5.28)	**0.004**
Lesion subtype				
Only leukoplakia^§^	Reference		Reference	
OSF/VH	3.73 (1.86-7.46)	**2.0 × 10^−4^**	3.99 (1.54-10.38)	**0.005**
Others^¶^	1.97 (0.83-4.63)	0.122	2.83 (1.06-7.56)	**0.038**
Treatment				
No medication/CO_2_ laser	Reference			
Biopsy/reconstruction	2.58 (1.1-6.06)	**0.030**		

Note: N = 734 (univariate); N = 730 (multivariable, due to missing data). *p*-values < 0.05 are in bold.

^#^ Covariates for maultivariabe analysis included lesion focality, lesion diversity, dysplasia, oral *Candidia* level, and lesion subtype.

^^§^^ Patients with leukoplakia (uniform) only.

^^¶^^ Patients not in the categories of ‘only leukoplakia’ or ‘OSF/VH’.

To identify independent predictors of MT, we conducted multivariable Cox regression modelling informed by both clinical relevance and statistical performance. Starting with an eight-variable model based on univariate associations, we applied stepwise selection using the Akaike Information Criterion (AIC) to aid in model refinement. This process resulted in a five-variable model including lesion multifocality, lesion diversity, *Candida albicans* burden, dysplasia, and lesion subtype (Table 2, right panel). Although AIC supported their inclusion, final model interpretation prioritised clinical importance, not model fit alone. To ensure the robustness of this multivariable model, we assessed multicollinearity among predictors using the variance inflation factor (VIF), all of which ranged from 1.08 to 2.65, well below the conventional threshold of 5, indicating no evidence of problematic collinearity (Supplementary Table S3).

In the final multivariable model, both high *Candida* burden (adjusted HR [aHR] = 2.84; 95% CI: 1.40–5.75; *P* = 0.004) and the lesions of OSF/VH (aHR = 3.99; 95% CI: 1.54–10.38; *P* = 0.005) remained independently associated with MT. As a sensitivity analysis, we repeated the multivariable model excluding patients with unknown *Candida* level (*N* = 543; Supplement Table S4). Results remained robust: high *Candida* burden (aHR = 2.86; 95% CI: 1.39–5.87; *P* = 0.004) and OSF/VH subtype (aHR = 5.01; 95% CI: 1.42–17.67; *P* = 0.012) continued to show strong associations. Although the reduced sample size led to wider confidence intervals, the directionality of effects was consistent for lesion multifocality and dysplasia. Collectively, these findings highlight high *Candida albicans* burden as an independent and biologically plausible risk factor for MT in patients with OPMD. Importantly, this association persisted across analytic approaches and was not driven by missing data, reinforcing its relevance in risk stratification.

### Interaction between *Candida albicans* and OPMD subtype on MT risk

**Figure 1.**
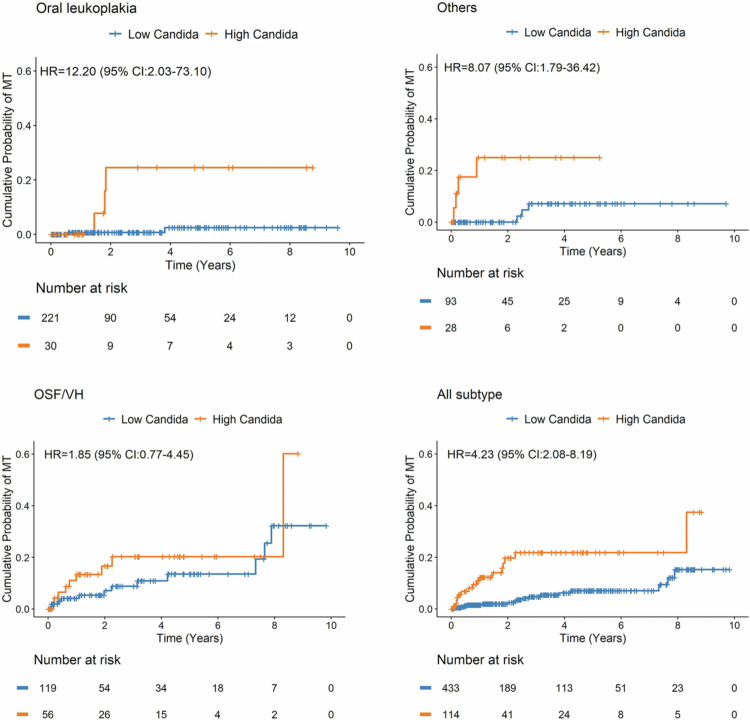
Divergent risk of MT by *Candida albicans* burden and OPMD subtype. Kaplan–Meier curves illustrating the cumulative probability of MT among patients with OPMD, stratified by *Candida albicans* burden (low vs. high) and lesion subtype. Analysis includes patients with available data on both variables (*n* = 547). The panels display survival trajectories for patients with leukoplakia (top left), OSF/VH (bottom left), other OPMD subtypes (top right), and the overall cohort (bottom right). Higher *Candida* burden was associated with increased risk in leukoplakia and other subtypes, while OSF/VH demonstrated elevated risk regardless of fungal load. HRs and 95% CIs were estimated using log-rank test for each comparison.

As shown in Figure 1, Kaplan–Meier curves revealed divergent patterns of accumulative incidence of MT stratified by both oral *Candida* burden and OPMD subtype. These findings prompted further investigation into potential effect modification. To formally test whether the impact of *Candida* load on transformation risk varied across OPMD subtypes, we extended the multivariable Cox regression model to include an interaction term between *Candida* burden and lesion subtype (Table 3). The model adjusted for lesion focality, lesion diversity, and dysplasia, and used patients with only leukoplakia and low *Candida* levels as the reference group.

**Table 3.** Multivariable Cox regression incorporating interaction between *Candida albicans* burden and OPMD subtype.

Variable	HR (95% CI)	*p*-value
Lesion focality		
Unifocal	Reference	
Multifocal	2.43 (1.12-5.26)	**0.026**
Lesion diversity		
Uniform	Reference	
Multiform	0.52 (0.23-1.15)	0.114
Dysplasia		
No	Reference	
Yes	2.86 (0.85-9.58)	0.09
No biopsy	0.59 (0.13-2.62)	0.502
Oral *Candida* level		
Low (0-50 CFU)	Reference	
High (>51 CFU)	13.83 (2.42-79.06)	**0.004**
Unknown	7.55 (1.54-37.11)	**0.014**
Lesion subtype		
Only leukoplakia^§^	Reference	
OSF/VH	13.67 (2.66-70.20)	0.002
Others^¶^	5.69 (0.91-35.36)	0.066
Interaction		
* Candida* low × leukoplakia	Reference	
* Candida* high × OSF/VH	0.11 (0.01-0.83)	**0.029**
* Candida* unknown × OSF/VH	0.27 (0.04-1.86)	0.161
* Candida* high × other lesions	0.5 (0.05-4.57)	0.567
* Candida unknown ×* other lesions	0.3 (0.03-2.8)	0.30

Note: N = 730 (as in multivariable model in Table 2). Statistically significant hazard ratios (95% CI not crossing 1.00) are presented in bold.

^^§^^ Patients with leukoplakia (uniform) only.

^**^¶^**^ Patients not in the categories of ‘only leukoplakia’ or ‘OSF/VH’.

In this interaction model, both high *Candida* burden (aHR = 13.83; 95% CI: 2.42–79.06; *P* = 0.004) and the presence of OSF/VH lesions (aHR = 13.67; 95% CI: 2.66–70.60; *P* = 0.002) were independently associated with significantly increased risk of MT. Notably, the interaction term between high *Candida* burden and OSF/VH subtype was negative and statistically significant (aHR = 0.11; 95% CI: 0.01–0.83; *P* = 0.029), suggesting an attenuated combined effect compared to the expected multiplicative risk. This sub-multiplicative property implies a less-than-additive, possibly antagonistic interaction: although each factor independently conferred substantial risk, their combination did not result in a proportionally higher hazard. Nonetheless, the estimated cumulative hazard for patients with concurrent OSF/VH and high *Candida* load remained markedly elevated (combined HR = 20.8) relative to the reference group.

Interaction terms involving other lesion types or unknown *Candida* status were not statistically significant, though effect directions were consistent with attenuation. Taken together, these findings indicate that the prognostic impact of *Candida albicans* is context-dependent and most pronounced in otherwise lower-risk lesions such as leukoplakia and possibly moderated by the underlying tissue microenvironment in higher-risk subtypes.

### Stratified Cox regression by OPMD subtype confirms context-dependent *Candida* risk

To further evaluate the subtype-specific impact of *Candida albicans* colonisation on MT, we conducted stratified Cox regression analyses within each major OPMD subtype (Table 4). Among patients with leukoplakia only (*n* = 344), high *Candida* burden was strongly associated with increased risk of MT (crude HR = 12.19; 95% CI: 2.12–70.12; *P* = 0.006). Even among those with unknown *Candida* status, the risk was moderately elevated (HR = 6.14; 95% CI: 1.27–29.61; *P* = 0.026).

**Table 4.** Stratified Cox regression analysis of *Candida albicans* burden and risk of MT by OPMD subtype.

OPMD subtype	N	*Candida* Level	HR (95% CI)	*p*-value
Only leukoplakia	344	Low	Reference	
		High	12.19 (2.12-70.12)	**0.006**
		Unknown	6.14 (1.27-29.61)	**0.026**
OSF/VH	224	Low	Reference 1.83 (0.76-4.44) 2.22 (0.88-5.57)	0.175 0.085
		High		
		Unknown		
Others	166	Low	Reference 8.16 (1.78-37.45)	**0.006**
		High		
		Unknown	2.73 (0.56-13.29)	0.219

Note: Stratified Cox models were fit separately within each OPMD subtype to assess the effect of *Candida* burden. HRs are relative to the reference group of low *Candida* burden within each subtype. *p*-values < 0.05 are in bold.

In contrast, among patients with OSF/VH group (*n* = 224), neither high *Candida* burden (HR = 1.83; 95% CI: 0.76–4.44; *P* = 0.175) nor unknown status (HR = 2.22; 95% CI: 0.88–5.57; *P* = 0.085) reached statistical significance. However, in the subgroup of other OPMD subtypes (*n* = 166), high *Candida* levels again conferred a significantly increased risk of MT (HR = 8.16; 95% CI: 1.78–37.45; *P* = 0.006), indicating that the influence of *Candida* burden extends beyond leukoplakia.

These stratified findings align with the interaction model, reinforcing that the prognostic impact of *Candida albicans* is context dependent. Its effect appears most pronounced in patients with lower-risk epithelial lesions, such as leukoplakia or other non-OSF/VH subtypes, where fungal overgrowth may serve as a co-factor in carcinogenesis. The absence of a significant association in the OSF/VH group may reflect distinct pathogenic mechanisms driving cancer risk in these high-risk lesions. Together, these results support the role of *Candida albicans* as a potential biomarker of malignant potential in select OPMD contexts.

### Association between *Candida* burden and OPMD subtypes

To further explore the relationship between *Candida* colonisation and specific OPMD subtypes, we performed chi-square test and logistic regression. Chi-square analysis showed that patients with OSF or VH lesions had a significantly higher prevalence of high *Candida* colonisation compared to those with other subtypes (*p* < 0.001, Supplementary Table S5). To further evaluate the direction and strength of this association, we performed two complementary logistic regression models (Supplementary Table S6). The first model assessed whether the presence of OSF/VH lesions predicted high *Candida* burden, while the second examined whether patients with high *Candida* colonisation had increased odds of OSF/VH lesions. Both models yielded concordant results, showing a consistent twofold association between OSF/VH lesions and high *Candida* levels (OR = 2.55, 95% CI: 1.65–3.94; *p* < 0.001). This suggests a statistically robust association between *Candida* colonisation and high-risk lesion types.

However, our stratified Cox regression and interaction analyses revealed that this association did not translate into an amplified MT risk in OSF/VH cases. Taken together, while OSF/VH and *Candida* colonisation are more likely to co-occur, their co-presence does not appear to drive MT synergistically. Rather, *Candida* colonisation may act as a context-specific biomarker of malignant potential, particularly in patients with lower-risk lesions such as leukoplakia, where its prognostic effect is most pronounced.

## Discussion

This multicenter, prospective cohort study demonstrates that high oral colonisation by *C. albicans* is independently associated with risk of MT in patients with OPMD. Notably, this risk is not uniform across lesion types but appears context-dependent, with the strongest association observed in patients with leukoplakia, lesions typically considered to carry a lower baseline risk. These findings suggest that *C. albicans* colonisation may serve as a biomarker of malignant potential in select epithelial contexts.

Our results align with prior experimental and observational studies implicating *C. albicans* in epithelial dysregulation and carcinogenesis via nitrosamine production, epithelial invasion, and pro-inflammatory signalling [18,22,30,31]. However, this is one of the first large-scale, prospective studies to quantify oral fungal burden and demonstrate its prognostic value using culture-based methods. The association between fungal load and MT remained robust after adjusting for conventional clinical predictors, including dysplasia and lesion subtype.

Stratified and interaction analyses further refined this relationship. High *C. albicans* was strongly predictive of MT in leukoplakia and ‘other’ OPMD subtypes, but not in OSF or VH. This attenuation in high-risk lesions may reflect their distinct histological architecture: subepithelial fibrosis and hyperkeratosis in OSF/VH may limit microbial penetration, immune surveillance, or epithelial response [32,33]. These structural barriers may restrict *Candida* invasion or blunt its pathogenic interaction with basal epithelial layers. In contrast, the relatively permeable epithelial structure of leukoplakia may be more susceptible to fungal invasion and carcinogenic byproduct exposure, amplifying fungal influence on dysplastic progression.

Interestingly, while alcohol use, betel quid chewing, and cigarette smoking are well-established carcinogens, none were independently associated with MT in multivariable models; this is consistent with prior research suggesting their role may be more prominent in carcinogenesis initiation than progression [12,34]. Our own prior murine study demonstrated that *C. albicans* alone was not tumorigenic but significantly enhanced tumour development when combined with arecoline and 4-nitroquinoline 1-oxide (4-NQO) [35]. These findings, supported by additional fungal–carcinogen models [36,37] support a promoter role for *C. albicans*, whereby fungal colonisation amplifies the effects of chemical carcinogens rather than acting as a standalone initiator.

We also observed that fungal burden correlated with certain lifestyle exposures, being higher among betel quid chewers and smokers, but lower in alcohol users (Supplementary Table S5). These trends may reflect mucosal disruption and inflammatory priming from tobacco and areca nut use [8,38,39], or microbiome-modulating effects of alcohol [40,41]. However, alcohol’s known role in acetaldehyde-mediated carcinogenesis precludes any interpretation of protection [23,42]. Similarly, the nonsignificant hazard ratio for current smoking likely reflects limited statistical power rather than a lack of biological effect.

Bidirectional logistic regression confirmed a strong association between OSF/VH lesions and high *C. albicans* burden. However, this does not imply that *Candida* plays a more dominant role in the malignant transformation among this subgroup compared to other OPMDs, as interaction analysis revealed that both high *Candida* burden and OSF/VH status were independently associated with elevated MT risk, and the interaction term between these two factors was attenuating (aHR = 0.11), indicating that their effects are additive rather than synergistic. These findings raise the intriguing possibility of a mutually reinforcing relationship: the altered stromal and epithelial environment characteristic of OSF/VH may favour persistent *Candida* colonisation, which in turn may contribute to chronic inflammation or lesion persistence. Yet, in these lesions, the interaction may contribute to the pathogenesis or persistence of OSF/VH without further amplifying their already elevated risk of malignant transformation. Therefore, caution is warranted when interpreting co-occurrence as evidence of interaction.

### Limitations

This study has several limitations that should be considered when interpreting the findings. As an observational cohort study, it cannot establish causal relationships between *Candida albicans* colonisation and malignant transformation. The assessment of fungal burden was based on a single baseline measurement, which may not fully reflect temporal dynamics in colonisation. Transient colonisation could have been misclassified as low burden, while persistent high-level colonisation may have gone undetected. Moreover, strain-level differences in *C. albicans*, which may influence pathogenicity, were not assessed.

Although participants with prior antifungal use were excluded at baseline, we did not systematically track antifungal or antibiotic treatments during follow-up. Other unmeasured factors such as oral hygiene practices, diet, systemic health, or immune status could also have influenced colonisation patterns or confounded the observed associations. While major known risk factors were adjusted for, the possibility of residual confounding remains.

Missing data is another consideration, as approximately 25% of participants lacked *Candida* burden data. To avoid bias related to likely non-random missingness, we treated these cases as a separate ‘unknown’ group rather than performing imputation. While this approach preserved the sample size, it may have influenced precision.

The threshold used to define ‘high’ *Candida* burden was derived from the observed data distribution within our cohort, which may limit generalisability to other populations. Nonetheless, sensitivity analyses using alternative CFU cutoffs yielded similar trends, suggesting internal robustness of the findings.

Precision in subgroup analyses was limited, particularly in the leukoplakia subgroup, where small event counts resulted in wide confidence intervals. These estimates should therefore be interpreted with caution.

Additionally, although the chromogenic agar medium can differentiate between *Candida* species [43], nearly all colonies in this study were morphologically consistent with *C. albicans*, and species-level confirmation was not performed. Lesion location and surface morphology were also not included in multivariable models due to the risk of overfitting with limited events.

Finally, the cohort was predominantly male and derived from a Taiwanese high-risk population, which may limit the applicability of findings to other demographic or geographic groups. Future research with serial fungal monitoring, strain-level identification, broader demographic inclusion, and more comprehensive microbiome analysis will be necessary to validate and refine these observations.

## Conclusions

In conclusion, high *Candida albicans* burden is an independent and context-dependent risk factor for malignant transformation in OPMD. Its strongest prognostic value was observed in clinically lower-risk subtypes such as leukoplakia, underscoring its potential as a subtype-sensitive biomarker. Incorporating fungal profiling into routine evaluation of OPMD may improve individualised risk stratification and inform targeted surveillance and prevention strategies in oral cancer care.

## Supplementary Material

Supplementary material Supplementary_Tables clean

Supplementary material Supplementary Methods
