# Supplementary material for: Candida albicans is a context-dependent risk factor for malignant transformation of oral precancer lesions: a prospective cohort study of 734 Taiwanese patients
Source: J Oral Microbiol. 2025 Dec 17;17(1):2598743. doi: 10.1080/20002297.2025.2598743 (PMC12716479; doi:10.1080/20002297.2025.2598743)
Supplement: Supplementary material — Supplementary_Tables clean [file ZJOM_A_2598743_SM3825.docx]

**Supplementary Tables**

Table S1. Distribution of oral swab count combinations and colonization patterns of *Candida albicans* among TWOPMD-1 participants.

Table S2. Comparison of baseline characteristics between included (N = 734) and excluded (N = 72) participants in the malignant transformation analysis.

Table S3. Variance inflation factor (VIF) analysis for multicollinearity assessment.

Table S4. Multivariable Cox regression analysis of factors associated with MT in OPMD (N=543).

Table S5. Association between *Candida* level and clinicopathological and behavioral variables.

Table S6. Association between *Candida* level and presence of OSF/VH: Logistic regression analyses.

**Table S1. Distribution of oral swab count combinations and colonization patterns of *Candida albicans* among TWOPMD-1 participants.** Each patient contributed swabs from all visible lesion sites and one non-lesion site (NL), with the number of swabs varying depending on lesion number. The table summarizes combinations of swab numbers and compares *C. albicans* colonization levels at non-lesion sites *versus* the highest lesion-site CFU (L_max_) observed for each patient. A five-level CFU scoring system was applied (Score 1: 0 CFU; Score 2: 1–20 CFU; Score 3: 21–50 CFU; Score 4: 51–100 CFU; Score 5: >100 CFU) to facilitate visualization and interpretation of colonization intensity, and to reduce over-interpretation of minor quantitative differences potentially affected by swabbing variability. This score-based representation is intended solely for descriptive comparison in this table and was not used for defining fungal burden in the main analyses.

| **Swab count pattern ^#^** | **No. of cases** | **Pattern of *Candida albicans* colonization levels*** | | |
| --- | --- | --- | --- | --- |
|  |  | **NL>L_max_** | **NL= L_max_** | **NL< L_max_** |
| 0 NL, 6 L | 1 | NA | NA | NA |
| 0 NL, 7 L | 2 | NA | NA | NA |
| 1NL, 0 L | 2 | NA | NA | NA |
| 1 NL, 1 L | 270 | 21 | 171 | 78 |
| 1 NL, 2 L | 177 | 19 | 114 | 44 |
| 1 NL, 3 L | 48 | 3 | 29 | 16 |
| 1 NL, 4 L | 30 | 2 | 19 | 9 |
| 1 NL, 5 L | 11 | 0 | 6 | 5 |
| 1 NL, 6 L | 2 | 0 | 1 | 1 |
| 1 NL, 7 L | 2 | 0 | 1 | 1 |
| 1 NL, 8 L | 2 | 0 | 1 | 1 |
| **Total** | 547 | 45 | 342 | 155 |

^#^ NL, non-lesion; L, lesion. A swab count of “1 NL, 3 L” indicates that the individual had one non-lesion swab and three lesion swabs.

* L_max_ : maximum level of *Candida albicans* detected among all lesion sites from the same individual.

**Table S2. Comparison of baseline characteristics between included (N = 734) and excluded (N = 72) participants in the malignant transformation analysis.** This table summarizes demographic, clinical, and behavioral characteristics of participants who were included versus those excluded from the MT analysis. Exclusions were due to early OSCC diagnosis within 30 days of enrollment or loss to follow-up before the first scheduled visit.

| **Variable** | **Excluded (%)** | | **Included (%)** |  | **Total (%)** | | |  |
| --- | --- | --- | --- | --- | --- | --- | --- | --- |
| Number of patients |  | |  |  | | |  |  |
|  | 72 (6.8) | | 734 (93.2) | 806 | | |  |  |
| Age (year) |  | |  |  | | |  |  |
| Mean (SD) | 52.06 (12.09) | | 52.31 (11.52) | 52.3 (11.5) | | |  |  |
| Follow-up (year) |  | |  |  | | |  |  |
| Mean (SD) | 0.14 (1.13) | | 2.43 (2.60) | 2.22 (2.58) | | |  |  |
| Gender |  | |  |  | | |  |  |
| Male | 63 (8.8) | | 655 (91.2) | 718 (89.8) | | |  |  |
| Female | 8 (9.8) | | 74 (90.2) | 82 (10.2) | | |  |  |
| Education |  | |  |  | | |  |  |
| Primary school | 34 (10.3) | | 297 (89.7) | 331 (41.4) | | |  |  |
| High school | 14 (10.2) | | 123 (89.8) | 137 (17.1) | | |  |  |
| College | 23 (6.9) | | 308 (93.1) | 331 (41.4) | | |  |  |
| Alcohol drinking |  | |  |  | | |  |  |
| Never | 8 (5.8) | | 129 (94.2) | 137 (17.2) | | |  |  |
| Occasional + Former | 29 (8.7) | | 304 (91.3) | 333 (41.7) | | |  |  |
| Current | 33 (10.1) | | 295 (89.9) | 328 (41.1) | | |  |  |
| Betel quid chewing |  | |  |  | | |  |  |
| Never | 15 (8.7) | | 157 (91.3) | 172 (21.6) | | |  |  |
| Occasional + Former | 34 (8.2) | | 381 (91.8) | 415 (52.1) | | |  |  |
| Ever | 21 (10) | | 189 (90) | 210 (26.3) | | |  |  |
| Cigarette smoking |  | |  |  | | |  |  |
| Never | 14 (11.8) | | 105 (88.2) | 119 (14.9) | | |  |  |
| Ever | 8 (5.6) | | 135 (94.4) | 143 (17.9) | | |  |  |
| Current | 48 (9) | | 487 (91) | 535 (67.1) | | |  |  |
| Lesion focality |  | |  |  |  | | |  |
| Unifocal | 37 (11.4) | | 288 (88.6) | 325 (40.4) | | |  |  |
| Multifocal | 33 (6.9) | | 446 (93.1) | 479 (59.6) | | |  |  |
| Lesion diversity |  | |  |  | | |  |  |
| Uniform | 45 (8.6) | | 479 (91.4) | 524 (65.3) | | |  |  |
| Multiform | 25 (9) | | 254 (91) | 279 (34.7) | | |  |  |
| Fibrosis | |  |  |  | | |  |  |
| No | 481 (94.9) | | 26 (5.1) | 548 (68.2) | | |  |  |
| Yes | 113 (91.1) | | 11 (8.9) | 255 (31.8) | | |  |  |
| (Continued on next page) | | | | | |  |  |  |
| (Continued) | | | | | |  |  |  |
| **Variable** | **Excluded (%)** | | **Included (%)** |  | **Total (%)** | | | |
| Dysplasia |  | |  |  | | |  |  |
| No | 13 (9.3) | | 127 (90.7) | 140 (17.5) | | |  |  |
| Yes | 25 (5.3) | | 447 (94.7) | 472 (59) | | |  |  |
| No biopsy | 32 (17) | | 156 (83) | 188 (23.5) | | |  |  |
| Oral *Candida* level |  | |  |  | | |  |  |
| Low (0-50 CFU) | 47 (9.8) | | 433 (90.2) | 480 (59.6) | | |  |  |
| High (>50 CFU) | 8 (6.6) | | 114 (93.4) | 122 (15.1) | | |  |  |
| Unknown | 17 (8.3) | | 187 (91.7) | 204 (25.3) | | |  |  |
| Major lesion subtype |  | |  |  | | |  |  |
| Only leukoplakia**^§^** | 26 (7) | | 344 (93) | 370 (45.9) | | |  |  |
| OSF/VH | 23 (9.3) | | 224 (90.7) | 247 (30.6) | | |  |  |
| Others**^¶^** | 23 (12.2) | | 166 (87.8) | 189 (23.4) | | |  |  |
| Treatment modality |  | |  |  | | |  |  |
| No medication/CO_2_ laser | 26 (13.8) | | 163 (86.2) | 189 (24.1) | | |  |  |
| Biopsy/reconstruction | 32 (5.4) | | 564 (94.6) | 596 (75.9) | | |  |  |

**^§^**Patients with leukoplakia (uniform) only.

**^¶^**Patients not in the category of “only leukoplakia” or “OSF/VH”.

**Table S3. Variance inflation factor (VIF) analysis for multicollinearity assessment.** This table reports the VIF values from a linear model constructed to assess multicollinearity among covariates included in the multivariable Cox regression analysis. A design matrix was generated using dummy variables for categorical predictors (variables in this table), and VIF values were computed from a linear model regressing a constant on these covariates. All VIF values are well below the commonly used threshold of 5, suggesting that multicollinearity is not a concern in this model.

| **Variable** | **VIF** |
| --- | --- |
| Lesion focality | 1.21 |
| Lesion diversity | 2.55 |
| Dysplasia | 1.89 |
| Dysplasia unknown | 1.87 |
| *Candida* level high | 1.12 |
| *Candida* level unknown | 1.08 |
| OPMD subtype-OSF/VH | 2.65 |
| Other OPMD subtype | 1.55 |

**Table S4. Multivariable Cox regression analysis of factors associated with MT in OPMD (N=543)**

| Variable | HR (95% CI) | P-value |
| --- | --- | --- |
| Lesion focality |  |  |
| Unifocal | Reference |  |
| Multifocal | 2.71 (0.99-7.37) | 0.051 |
| Lesion diversity |  |  |
| Uniform | Reference |  |
| Multiform | 0.54 (0.20-1.46) | 0.225 |
| Dysplasia |  |  |
| No | Reference |  |
| Yes | 2.68 (0.59-12.11) | 0.200 |
| No biopsy | 0.50 (0.07-3.69) | 0.499 |
| Oral *Candida* level |  |  |
| Low (0-50 CFU) | Reference |  |
| High (>51 CFU) | 2.86 (1.39-5.87) | **0.004** |
| Lesion subtype |  |  |
| Only leukoplakia^§^ | Reference |  |
| OSF/VH | 5.01 (1.42-17.67) | **0.012** |
| Others**^¶^** | 4.60 (1.24-17.12) | **0.023** |

Note:

This model includes patients with complete data and known *Candida* levels (N = 543).

^§^Patients with leukoplakia (uniform) only.

**^¶^**Patients not in the categories of “only leukoplakia” or “OSF/VH”.

P-values <0.05 are in bold.

**Table S5. Association between *Candida* level and clinicopathological and behavioral variables.** This table summarizes the distribution of demographic, lifestyle, and clinical characteristics across patients with low and high *Candida* levels. Analysis is limited to individuals with known *Candida* status (N = 547).

| **Variable** | **Oral *Candida* level** | | **P-value** |  |
| --- | --- | --- | --- | --- |
|  | **Low (0-50 CFU) (%)** | **High (>50 CHU) (%)** |  |  |
| Age (year) |  |  | 0.468 |  |
| Mean (SD) | 52.33 (11.26) | 53.18 (11.11) |  |  |
| Gender |  |  | **0.033**  0.033 |  |
| Male | 383 (77.7) | 110 (22.3) |  |  |
| Female | 45 (91.8) | 4 (8.2) |  |  |
| Education |  |  | 0.114 |  |
| Primary school | 203 (91.9) | 18 (8.1) |  |  |
| High school | 87 (97.8) | 2 (2.2) |  |  |
| College | 219 (94.8) | 12 (5.2) |  |  |
| Alcohol drinking |  |  | **0.010** |  |
| Never | 72 (68.6) | 33 (31.4) |  |  |
| Occasional + Former | 177 (79.7) | 45 (20.3) |  |  |
| Current | 178 (83.2) | 36 (16.8) |  |  |
| Betel quid chewing |  |  | **0.012** |  |
| Never | 97 (88.2) | 13 (11.8) |  |  |
| Occasional + Former | 219 (78.5) | 60 (21.5) |  |  |
| Current | 111 (73) | 41 (27) |  |  |
| Cigarette smoking |  |  | **0.005** |  |
| Never | 69 (93.2) | 5 (6.8) |  |  |
| Former | 73 (78.5) | 20 (21.5) |  |  |
| Current | 285 (76.2) | 89 (23.8) |  |  |
| Lesion focality |  |  |  |  |
| Unifocal | 176 (83.4) | 35 (16.6) | 0.067 |  |
| Multifocal | 257 (76.5) | 79 (23.5) |  |  |
| Lesion diversity |  |  | **5.64×10^-5^** |  |
| Uniform | 295 (84.5) | 54 (15.5) |  |  |
| Multiform | 137 (69.5) | 60 (30.5) |  |  |
| Fibrosis |  |  | **0.001** |  |
| No | 299 (83.5) | 59 (16.5) |  |  |
| Yes | 133 (70.7) | 55 (29.3) |  |  |
| Dysplasia |  |  | **0.026** |  |
| No | 80 (84.2) | 15 (15.8) |  |  |
| Yes | 245 (75.2) | 81 (24.8) |  |  |
| No biopsy | 104 (85.2) | 18 (14.8) |  |  |
| (continued on next page) | | | |  |
|  |  |  |  |  |
| (continued) | | | |  |
| **Variable** | | **Oral *Candida* level** | | **P-value** |
|  |  | **Low (0-50 CFU) (%)** | **High (>50 CHU) (%)** |  |
| Lesion subtype | |  |  | **2.73×10^-6^** |
| Only leukoplakia**^§^** | 221 (88) | 30 (12) |  |  |
| OSF/VH | 119 (68) | 56 (32) |  |  |
| Others**^¶^** | 93 (76.9) | 28 (23.1) |  |  |
| Treatment |  |  |  |  |
| No medication/CO_2_ laser | 111 (85.4) | 19 (14.6) | 0.054 |  |
| Biopsy/reconstruction | 318 (77) | 95 (23) |  |  |

**^§^**Patients with leukoplakia (uniform) only.

**^¶^**Patients not in the categories of “only leukoplakia” or “OSF/VH”.

**Table S6. Association between *Candida* level and presence of OSF/VH: logistic regression analyses.** Model 1 examines the odds of high *Candida albicans* colonization given the presence of OSF or VH. Model 2 reverses the outcome and predictor, examining the odds of OSF/VH given high *Candida albicans* colonization. Both models were performed on the same cohort (N = 547) and adjusted only for univariate associations.

| Model | Outcome | Predictor | Estimate | OR | 95% CI | P-value |
| --- | --- | --- | --- | --- | --- | --- |
| 1 | High *Candida* level | OSF/VH | 0.94 | 2.55 | 1.65-3.94 | **1.5×10^-5^** |
| 2 | OSF/VH | High *Candida* level | 0.94 | 2.55 | 1.65-3.94 | **1.5×10^-5^** |
