# Supplementary material for: Candida albicans is a context-dependent risk factor for malignant transformation of oral precancer lesions: a prospective cohort study of 734 Taiwanese patients
Source: J Oral Microbiol. 2025 Dec 17;17(1):2598743. doi: 10.1080/20002297.2025.2598743 (PMC12716479; doi:10.1080/20002297.2025.2598743)
Supplement: Supplementary material — Supplementary Methods [file ZJOM_A_2598743_SM3826.docx]

**Supplementary Methods**

**Colony RT-PCR for validation of *Candida albicans* presence**

To confirm the presence of *Candida albicans* colonies grown on chromogenic agar, a species-specific colony RT-PCR was performed, adapted from Guiver et al. (reference #24 in the main text). Colonies were first subcultured on Sabouraud dextrose agar at 30°C overnight. A single colony was then suspended in 1 mL of sterile water and adjusted to an optical density of 0.1 at A_650_. DNA extraction was performed by mixing 100 μL of the suspension with 1 mL of DNAzol reagent (Thermo), followed by vortexing and incubation at room temperature for 10 minutes. DNA was precipitated by adding 1 mL of ethanol and centrifuged at 12,000 × g for 10 minutes. The resulting pellet was washed with 1 mL of ethanol and resuspended in 50 μL of sterile water. PCR amplification targeted a 108 bp fragment within the *ITS2* (internal transcribed spacer 2) region, using the following primers:

- Forward: 5’-GGGTTTGCTTGAAAGACGGTA-3’

- Reverse: 5’-TTGAAGATATACGTGGTGGACGTTA-3’

PCR products were analyzed by electrophoresis on a 2% agarose gel, stained with ethidium bromide, and visualized under UV light. A distinct band at 108 bp confirmed the identity of *C. albicans.*
